# Closed Structural Rhinoplasty

**DOI:** 10.1007/s00266-025-05122-1

**Published:** 2025-09-16

**Authors:** Richard P. Clark, Matthew M. Farajzadeh, Granger B. Wong

**Affiliations:** 1 Private Practice, Sacramento, CA USA; 2 Division of Plastic and Reconstructive Surgery, University of California Davis, 22 Quail Point Place, Carmichael, Sacramento, CA USA

**Keywords:** Rhinoplasty, Closed, Endonasal, Structural

## Abstract

**Background:**

Closed rhinoplasty had initially been relegated to a reductive procedure that did not provide access to place structural sutures for tip support. Despite the influence of closed rhinoplasty advocates Sheen, Constantian, Guerrerosantos, Bravo, Kamburoglu, Tezel, Ersoy, Cakir, Valdivia and others who have produced excellent results, most surgeons prefer the open approach. The scar resulting from open rhinoplasty is considered a relatively innocuous necessity to achieve sophisticated results.

**Methods:**

Endonasal methods to replicate the tip suturing techniques of open rhinoplasty were developed over 39 years by the senior author. This paper describes, and supplemental videos demonstrate, the tip suture passing and grafting techniques through the closed approach. To evaluate the frequency of columella scar negative consequences, a survey of ASPS members was done.

**Results:**

Videos and photographic examples demonstrate the success of these original closed rhinoplasty suture techniques. In a survey of ASPS surgeons, 11% of respondents report that over 25% of patient’s scars were noticeable at intimate distances.

**Conclusion:**

Tip restructuring with permanent sutures can be readily done via the delivery technique of closed rhinoplasty without creating a columella scar that may be problematic.

**Level of Evidence V:**

This journal requires that authors assign a level of evidence to each article. For a full description of these Evidence-Based Medicine ratings, please refer to the Table of Contents or the online Instructions to Authors https://www.springer.com/00266.

**Supplementary Information:**

The online version contains supplementary material available at 10.1007/s00266-025-05122-1.

## Introduction

The authors of the seminal book “Open Structure Rhinoplasty” stated that closed rhinoplasty was essentially a reductive procedure and that the open approach is required to afford the exposure to reshape or restructure the nose [1]. The authors were referring to the tip, since their approach to the upper two-thirds of the nose was no different than the traditional endonasal approach [1]. The initial paradigm shift to open rhinoplasty was because of the tip restructuring with medial crural struts and sutured tip grafts. Despite the influence of closed rhinoplasty advocates Sheen, Constantian, Guerrerosantos, Bravo, Kamburoglu, Tezel, Ersoy, Cakir, Valdivia and others who have produced excellent results, most surgeons prefer the open approach and many have never been trained in closed rhinoplasty. As recent as 2018, respected rhinoplasty surgeon Rollin Daniel expressed the opinion that the current sophisticated tip suturing techniques are “either impossible or technically challenging via a closed approach” [2]. Our paper, representing the senior author’s 39-year experience with rhinoplasty, intends to show that tip restructuring with intercrural and interdomal sutures, septal extension grafts [SEGs] and fixated tip grafts are not only possible through the endonasal approach, but, often, not more difficult. The techniques herein described consist of passing sutures under the tip skin to comfortably work separately through each nostril. They can be done routinely, and a scar on the columella is not necessary.

Sheen argued that through hidden incisions of closed rhinoplasty, strategic trimming, reshaping and repositioning of the lower lateral cartilage, with or without tip graft augmentation, could nicely improve tip shape [3]. The Sheen (shield) and Peck (onlay)tip grafts were held in place by a tight pocket and external taping and then sticky fibrin eventually became a scar adhesion immobilizing the tip grafts [4,5]. Nonetheless, tip grafts could displace and excisional reduction techniques at times weakened the alar cartilages, resulting in tip widening and loss of projection [6, 7]. Rhinoplasty tip support and shape took a quantum leap with columellar struts and alar cartilage and tip graft suture techniques performed via an open approach introduced by otolaryngologist/facial plastic surgeons: Anderson, Johnson and Toriumi [1,8]. Brilliant surgeons including Gunter, Rohrich, Gruber and Daniel carried the torch of open rhinoplasty to plastic surgeons throughout the world [9–11]. These surgeons showed excellent results that seemingly could only be accomplished via an open approach and, thus, justified the columella scar.

**Fig. 1:**
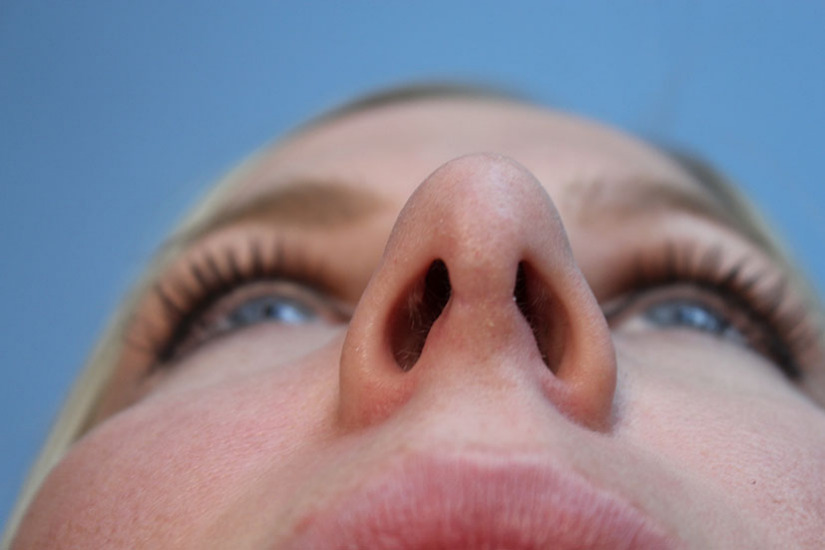
A photo of an open rhinoplasty scar that was problematic on casual observation and was particularly noticeable at intimate distances.

Jack Gunter defended the columella scar by stating that the scar was not noticeable upon “casual observation” or at “conversational distances” [12]; yet certainly most people would prefer their rhinoplasty to remain confidential and that a scar not be noticeable at intimate distances. One could argue that, since the scar resulting from the open approach is not always innocuous and may be notched, wide or discolored (Fig. 1), the closed approach may be preferable if equivalent results can be achieved.

We conducted a survey of ASPS members to garner an opinion about columella scars (Table 1). In an era of “Preservation Rhinoplasty,” it would seem appropriate to preserve the natural beauty of the fluid smooth curve of an uninterrupted columella.

The open view of undisturbed tip anatomy has value, but knowledge of anatomy and careful pre-op scrutiny of visual and palpable anatomy may obviate the need to lift the skin off the tip. Our standard endonasal approach is the classic delivery technique which at one time was taught to all otolaryngology and plastic surgery residents. It is best taught in a training atmosphere, but it is not complicated and consists of the following maneuvers:

1. An intercartilaginous incision is made which continues medially just caudal to and around the anterior septal angle. It then connects to a full transfixion incision which transects through both leaves of the membranous septum while hugging the caudal end of the cartilaginous septal plate. The septum and dorsum and midvault are accessed through these incisions and altered as needed.
2. A rim incision is made that is similar to an open rhinoplasty incision but without the horizontal component across the visible columella. The paracolumellar portion of the incision is carried posteriorly, beyond that of an open approach, toward the medial crura feet, and thus is made adequately long to allow dissection of the alar cartilages from restrictive attachments. The dissection is done with a Stevens or Tenotomy scissors, either in a subperichondrial or supraperichondrial plane, in a cephalic direction between the overlying skin envelope and the domes and the lateral crura, until it reaches the intercartilaginous incision. Dissection from the paracolumellar incision to the transfixion incision proceeds between the domes and middle crura and at least the anterior portion of the medial crura, which are separated from each other. This dissection leaves the vestibular skin attached to the alar complex and allows for a healthy bipedicle chondromucosal flap and a “bucket handle” delivery of each lower lateral cartilage (LLC) through each nostril for visualization and reconstruction. The rim incision is preferred over a marginal incision because it provides a wider lateral leg of the bipedicle flap. This combination of viewing the alar cartilages both in situ, and also in the delivered position, is quite sufficient to verify the problems that are anticipated by a thoughtful pre-op evaluation. This delivery of most of the alar complex readily allows such common transformations as domal and lateral crural reshaping and repositioning (Video 1).

However, the task of reshaping the alar cartilage is not as daunting as the quandary of how, without the exposure of open rhinoplasty, to preserve or increase tip support (with or without de-rotation) and how to secure tip grafts. Actually, the simple act of delivering each alar cartilage typically involves lysing the interdomal and intercrural ligaments as well as the longitudinal and vertical scroll ligaments, which will weaken tip support unless reconstructed [13]. Therefore, solutions to achieving this reconstruction and gain tip support are paramount and are described in the text and videos that follow.

## Methods

### Tip Support Restructuring with Closed Rhinoplasty Exposure

The structural support of the tip provided by the natural tripod may be sufficient after the LLC’s are trimmed or otherwise altered and reshaped, but further tip support is sometimes needed to maintain desired tip projection. A main impetus for the open approach is to increase tip support by placing sutures which bring the middle crura and the domes together, with or without an intervening strut or Septal Extension Graft (SEG) to maximize strength and control of tip position. However, the closed approach also allows placement of these Intercrural and Interdomal sutures and readily allows the use of the ubiquitous SEG to control projection and rotation of the tip. Additionally, one can readily secure tip support while simultaneously correcting a hanging columella with the Sandwich (tongue-in-groove) technique via the closed approach.

If the patient’s nostrils are large enough, and the soft tissue envelope is sufficiently supple, to allow both alar complexes to be delivered through one nostril, the intercrural and interdomal sutures and intervening grafts can be placed through that one nostril as elegantly shown by Valdivia [14]. The first part of Video 2 demonstrates such a “full” delivery through one nostril. However, working through both nostrils using the suture passing techniques herein described is applicable to all noses and is described as follows.

#### Intercrural Sutures via Closed Approach

Each domal complex is delivered through each nostril allowing easy access for placing sutures. A 4-0 nylon suture can be placed into and out of the delivered right middle cru and simply passed from that right nostril under the previously elevated tip skin and retrieved through the left nostril where the suture is secured to the delivered left middle cru. To ensure domal projection symmetry, a caliper is used to record the distance from the dome of each pass of the needle as it enters and exits the right middle cru and then the distances are duplicated for the left middle cru needle passage. These intercrural sutures can be placed with or without an intervening medial or middle crural strut or SEG. Now the tail of the 40 nylon is passed from the right nostril, under the tip skin and out the left nostril. Cinching of this intercrural suture is routinely done with both domes delivered back under the tip skin (Video 2, Video 4).

**Fig. 2:**
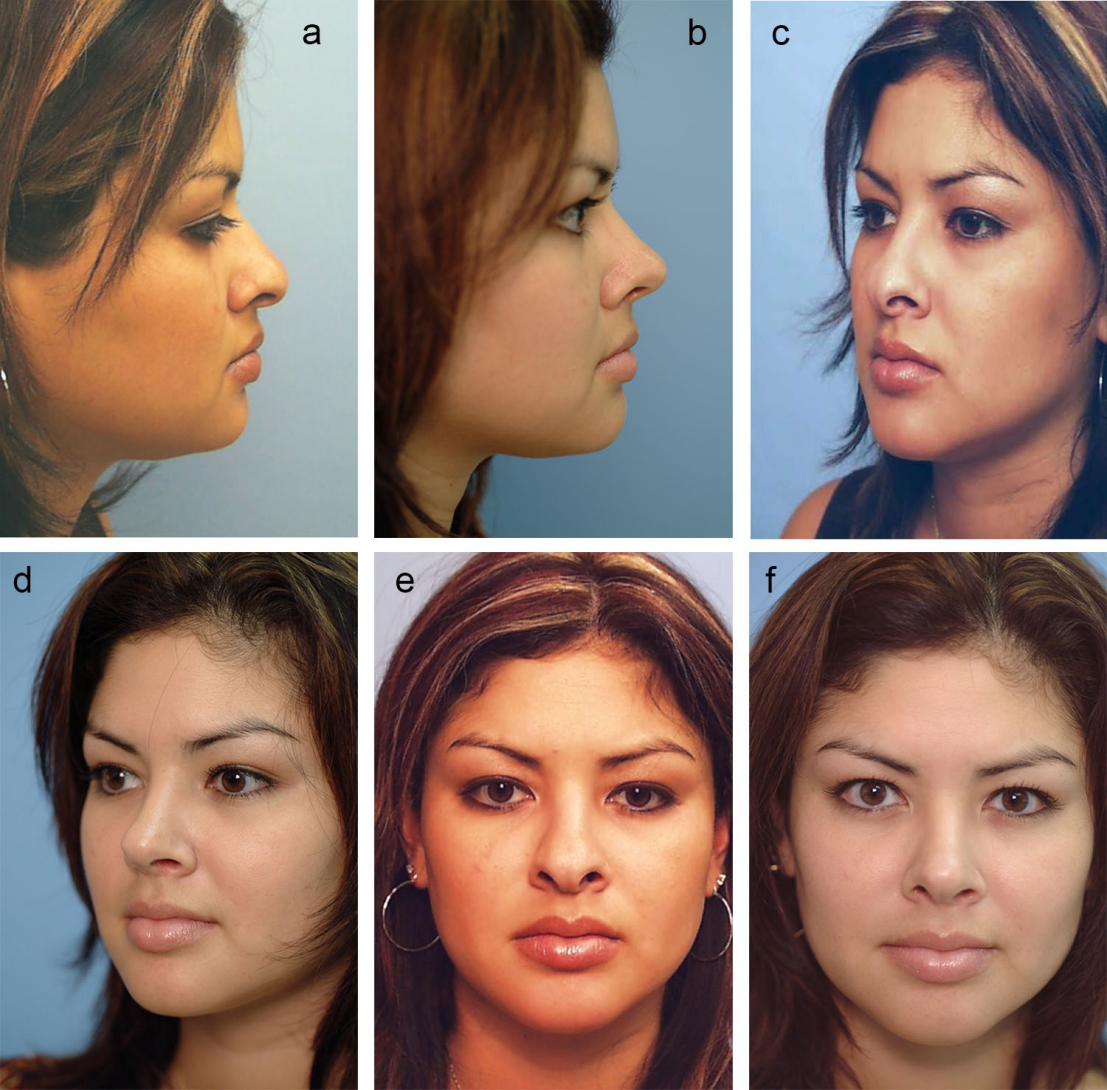
**a** Pre-op lateral view photo Hispanic patient. **b** Six-month post-op lateral view. Closed rhinoplasty included intercrural and interdomal sutures, lateral steal and infradomal grafts to increase projection and reshape the tip. A septal extension graft was placed (technique as described herein) to the left side of the septum to prevent excessive tip rotation and support tip projection. **c** Pre-op oblique view. **d** Post-op oblique view. **e** Pre-op AP view. **f** Post-op AP view

**Fig. 3:**
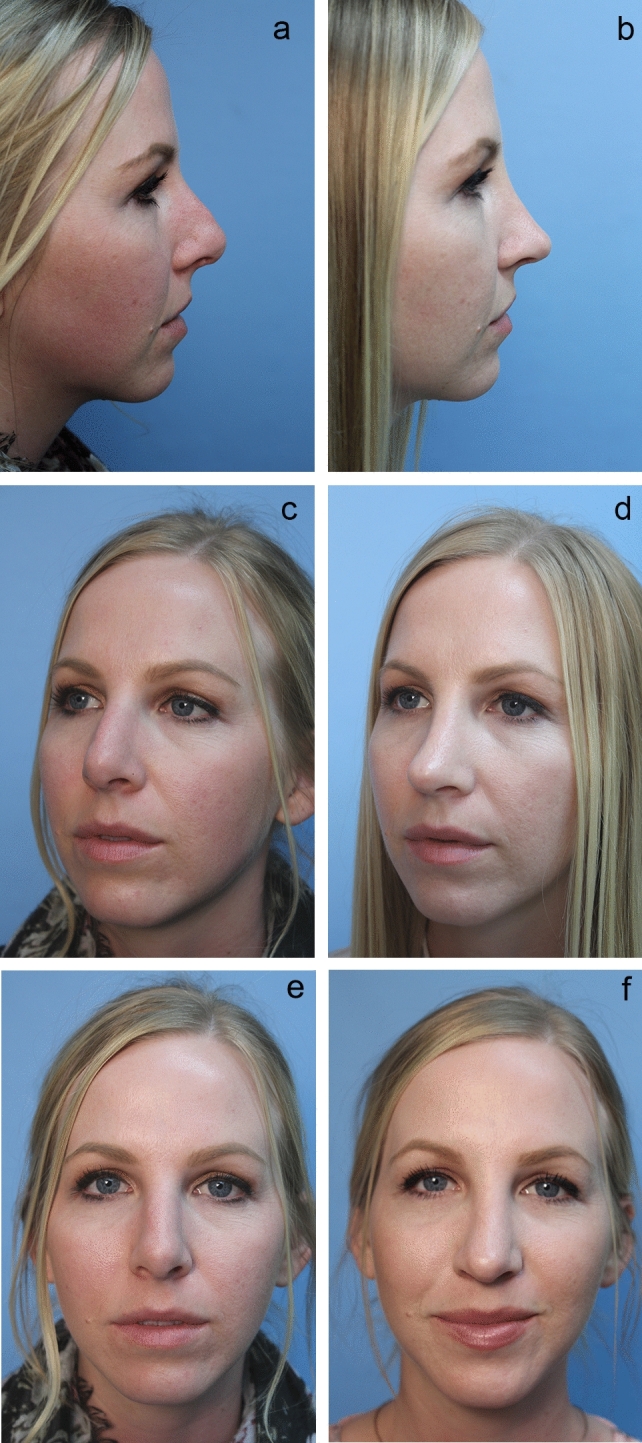
**a** Pre-op lateral view of patient unhappy with open rhinoplasty. (see columella scar in Fig 1). **b** Six-month post-op lateral view. Closed rhinoplasty included “Bowsprit” SEG and Peck and lobular tip grafts and chin augmentation. **c** Pre-op oblique view. **d** Post-op oblique view. **e** Pre-op front view **f** Post-op front view

**Fig. 4:**
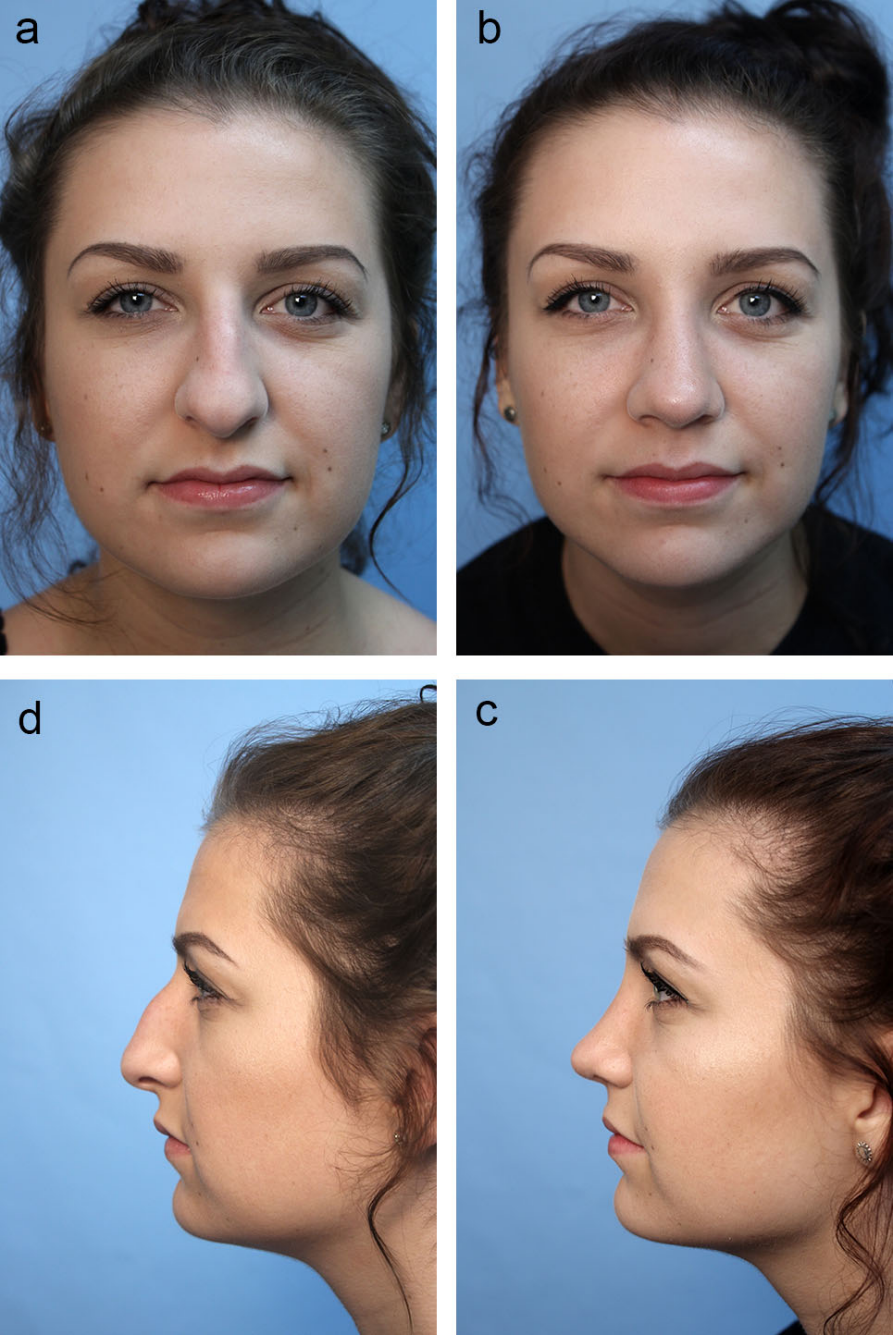
**a** Pre-op lateral view. **b** One-year post-op lateral view. Closed rhinoplasty featuring Tongue-in Groove [Sandwich] procedure and included infradomal grafts, intercrural suture of middle crural strut, interdomal suture and Peck and lobular tip grafts. **c** Pre-op front view. **d**. Post-op view [slight over correction of hanging columella]

**Fig. 5:**
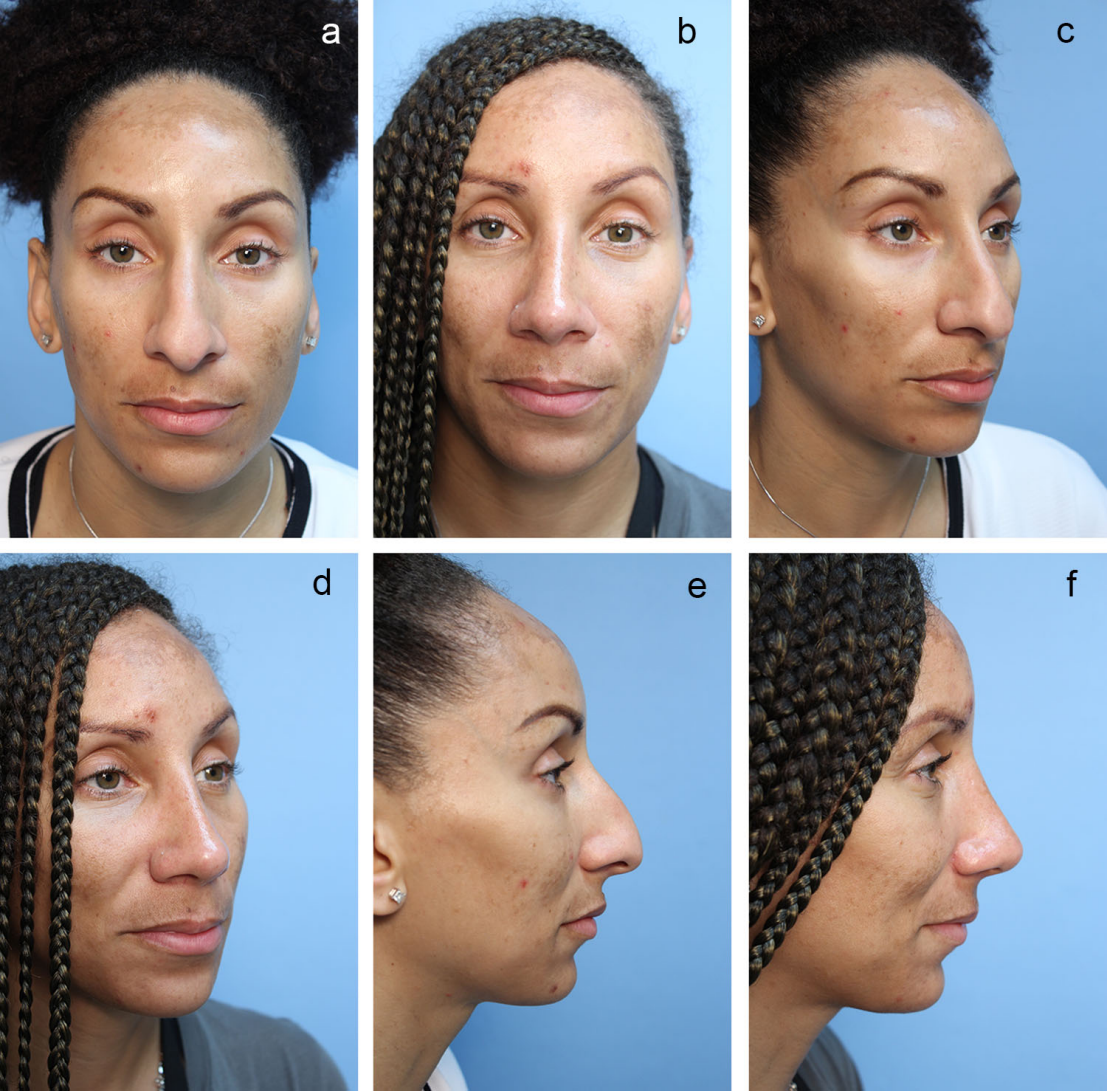
**a** Pre-op front view. **b** Six-month post-op front view. Black female with closed rhinoplasty including hump reduction, middle crural strut, infradomal grafts and Sandwich [tongues–ingroove] technique onto straight septum. Did not desire nostril reduction. **c** Pre-op oblique view. **d** Post-op oblique view. **e** Pre-op lateral view. **f** Post-op lateral view

**Fig. 6:**
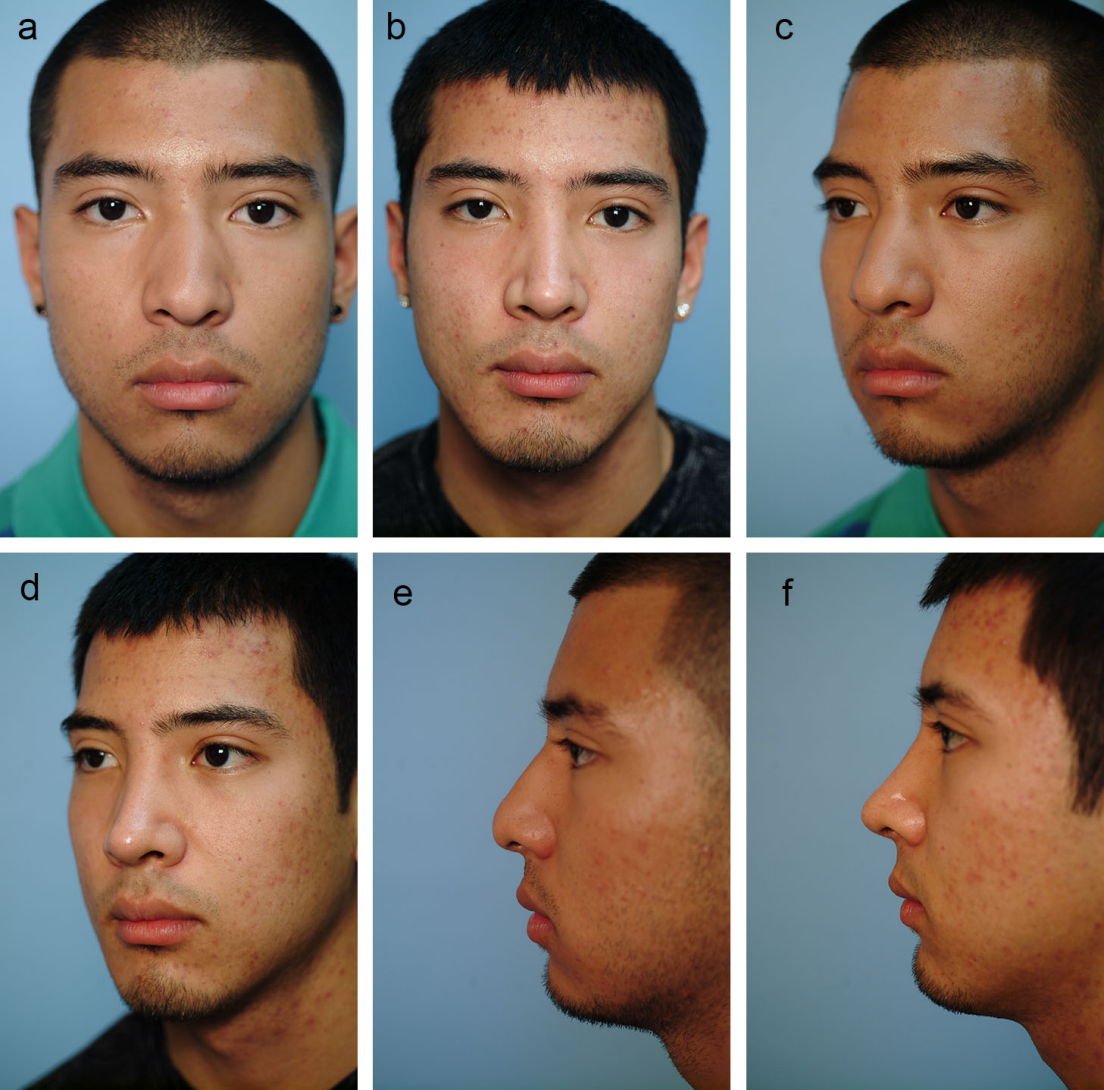
**a** Pre-op front view. **b** Six-month post-op front view. Asian male with closed rhinoplasty including dorsal rasping and osteotomies, intercrural SEG to side of septum, interdomal sutures, Peck and lobular grafts and chin augmentation. **c** Pre-op oblique view. **d** Post-op oblique view. **e**. Pre-op lateral view. **f**. Post-op lateral view

**Fig. 7:**
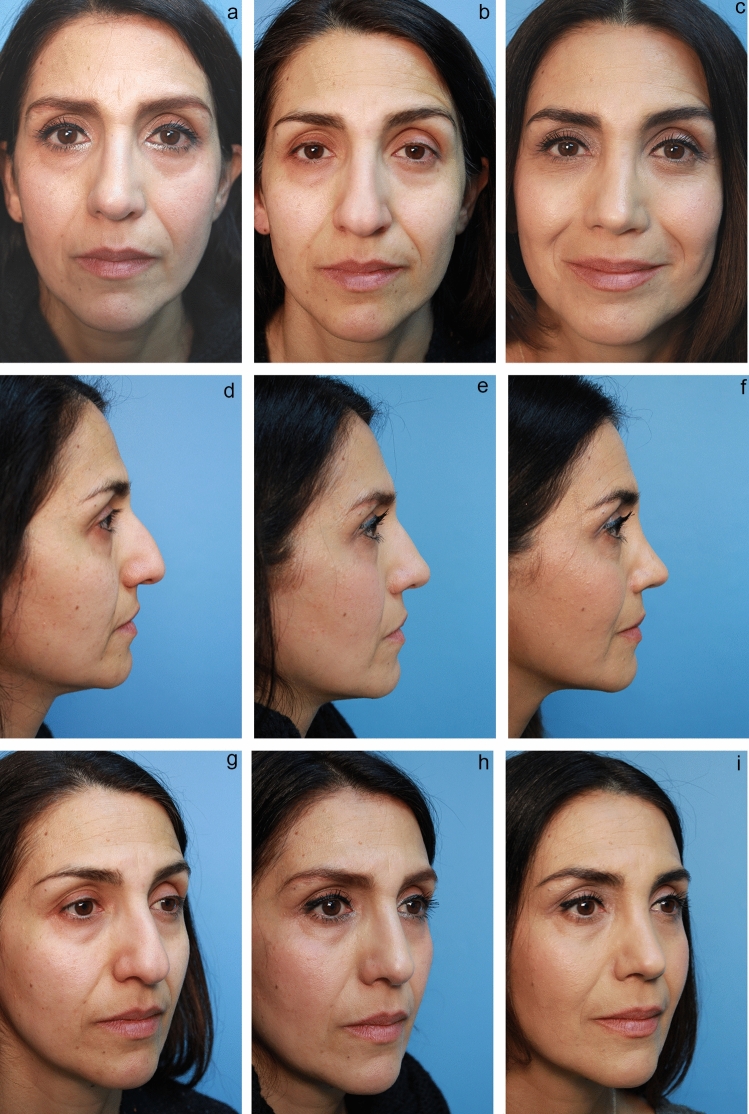
**a** Pre-op front view of middle eastern patient c/o hump and that tip is too big. **b** 18 month post-op open rhinoplasty done elsewhere. Although there is conservative reduction of size, she is unhappy that it is still too “big” and she wants a more feminine nose. **c** 7 months postop closed revision including osteotomies to close open roof and SEG and Peck graft using the techniques herein described. **d** Pre-op lateral view. **e** 18 months post-op open rhinoplasty. **f** 7 months post op closed revision creating the slight "scooped" bridge she desired. **g** Pre-op Oblique view. **h** 18 month post-op open rhinoplasty. **i** 7 months post-op closed secondary rhinoplasty

Since it is important to place interdomal sutures [described below] prior to tying down the intercrural suture, both ends of the intercrural suture, which are extruding from the left nostril, should be temporarily taped to the left of the nose and out of the way. Once the interdomal suture has been placed, the intercrural suture is cinched down through the left nostril bringing middle crura together (Figs. 2, 3, 4, 5, 6, and 7).

#### Interdomal Sutures via Closed Approach

After placing the intercrural suture as described above, one must not tie down the intercrural suture until the interdomal suture has been placed. Rather, one must tape down the intercrural suture ends out of the way to avoid entangling the two separate 4-0 nylon sutures. The interdomal 4-0 nylon suture can then be placed from medial to lateral through the cephalic half of the right dome. Then the swaged end of the suture needle is simply passed from that right nostril under the previously elevated tip skin and delivered through the left nostril. Now the needle is passed from lateral to medial through the cephalic half of the left dome and the needle is passed back under the elevated tip skin and retrieved out the right nostril. Both ends of the suture are now coming out the right nostril and should be taped out of the way on the right side of the nose. At this point, attention turns to the intercrural suture protruding from the left nostril, and it is firmly tied down. Returning to the right, the interdomal suture is more gently cinched to bring the domes together at their cephalic aspect to mimic the angle of domal separation and to pull the domes slightly more anterior to any middle crural strut or SEG [15] (Videos 3 and 4; Figs. 2, 3, 4, 5, 6, and 7).

#### Septal Extension Graft (SEG) via Closed Approach

**Fig. 8:**
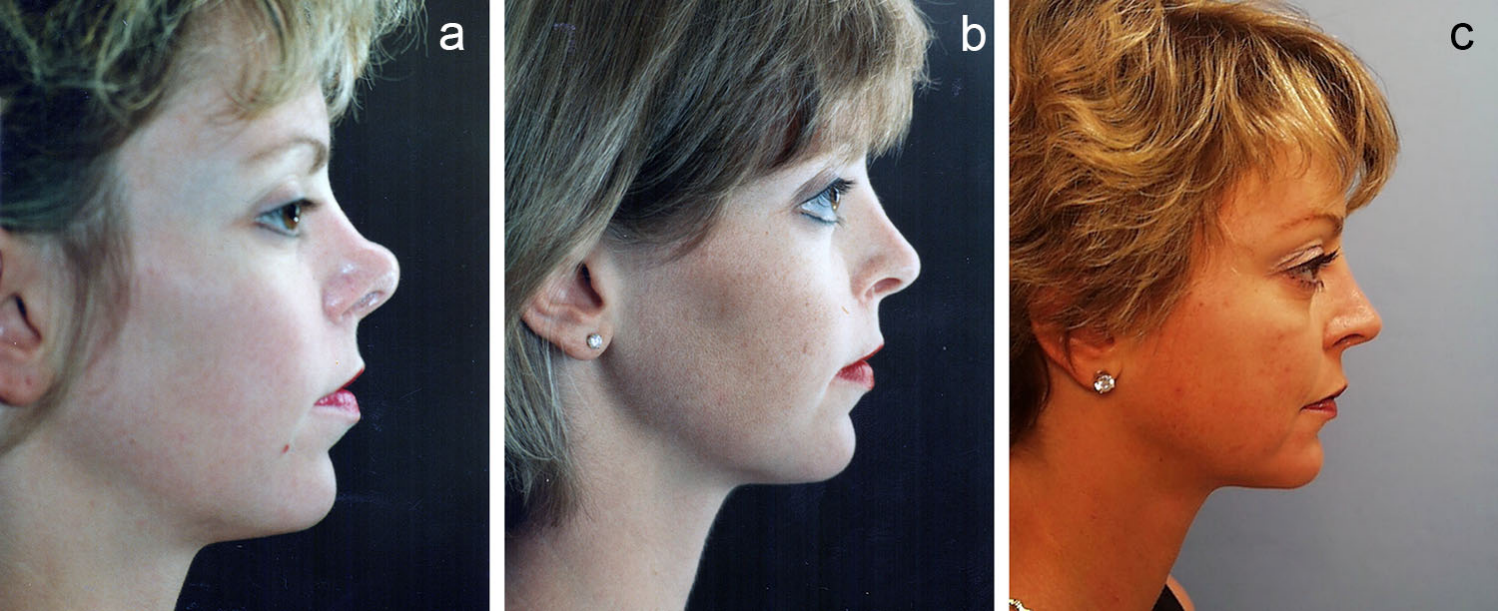
**a** Pre-op lateral view of congenital short over-rotated nose. **b** Six-month post-op lateral view. Closed rhinoplasty included septal extension graft attached to caudal end of septum 40 nylon sutures placed with figure of eight to prevent overlap. **c** Twenty-year post-op result. Patient had touch up at 1-year post-op to place conchal cup composite grafts to lower alar rims

Septal extension grafts (SEGs) are overall more useful than medial crural columellar struts which “do not prevent cephalic rotation of the tip-lobule complex and therefore offer no control of length unless attached to the caudal septum” [16–18]. SEGs are very useful to control the tip rotation and projection and, in the senior author’s practice, have completely replaced the medial crural strut of open rhinoplasty. Septal extension grafts were first utilized to correct natural or iatrogenic short noses turned up with too much “Miss Piggy” nostril show. At the 1994 annual meeting of the California Society of Plastic Surgery, Dr. Clark presented his technique to lengthen short noses by using two “figure of eight” 4-0 nylon sutures to secure a “septal lengthening graft” to the caudal end of the septum (Fig. 8). A similar concept was concurrently described by Gruber and later popularized by Byrd as septal extension grafts [16,19].

Over the years, the senior author found it more practical to first secure the SEG to each middle crura with the same technique that I describe above for intercrural sutures that include middle struts or SEGs (Videos 2 and 4). Although autograft rib is popular, one can usually harvest the SEG from the septum or concha and recently the senior author has had a good result using fresh frozen allograft rib cartilage. Once the SEG is secured to both middle cru/domal complexes, it is then attached to either the right or left side of the quadrilateral septal plate. Which side of the septum depends on any deviation of the caudal end of the septum and the curvature [if any] of the SEG, with the goal to achieve a non-deviated tip with proper projection and rotation (Video 4; Figs. 2, 6, and 7)

Occasionally, if the caudal septum is straight, one can suture two relatively weak cartilage SEGs together to increase their strength and, the anterior-caudal end of the two-tiered graft is secured to the middle crura. The cephalic end is splayed out and sandwiched over both sides of the septum in the resemblance of a ship’s “Bowsprit” (Video 5; Fig. 3).

#### “TONGUE-in-GROOVE” or “Sandwich” SUTURES via Closed Approach

If the septum is straight but is too long, and the columella encroaches on the upper lip with too much nostril show, it can be corrected by using a Sandwich technique, which is also described as a “tongue-in-groove” [20]. In order to ensure a concurrent strong and projected tip, only the medial crura are sandwiched and an intercrural suture with or without a middle crural strut projects the tip. This avoids too much cephalic tip rotation and operates with the same principles achieved with a modification described by Daniel [13].

Using tenotomy scissors dissection proceeds from the transfixion incision caudally and a space is separated between the two leaves of the membranous septum and the medial crura which will then be sandwiched superiorly onto the caudal septum. The 4-0 nylon suture placement is unique but simple and very effective. A straight or straightened P-3 needle is passed in a caudal direction from the right transfixion incision under the skin on the right side of the membranous septum and columella and brought out the columella portion of an extended rim incision. The needle is placed back in the same opening and passed across the columella either in front of or through both medial crura and brought out the columella extension of the left rim incision staying in the same horizontal plane. The needle is then placed back into the same opening and passed cephalically under the columella skin and membranous septum until it exits from the left side transfixion incision. The columella and tip can be brought up and back and sandwiched onto the caudal end of the septum. Proper retraction and projection are adjusted until the appearance is right and then the nylon is secured to the septal plate. This sounds complicated and therefore requires visualization (Video 6; Figs. 4 and 5)

### Tip Grafting with Closed Rhinoplasty Exposure

Tip grafts can be secured soundly via an open approach and, therefore, can be large shield type grafts that contribute significantly to projection. On the other hand, the closed rhinoplasty surgeon should more often depend on creating strong tip projection mostly by using the patient’s alar cartilage that are strengthened with intercrural sutures plus or minus intervening struts and septal extension grafts. Strengthening the domes with infradomal grafts may also contribute to projection (Video 7; Figs. 2, 4, 5) [21]. The Peck type onlay tip grafts (usually slightly morselized to soften) are used to “fine-tune” the projection and contour. If the tip graft is not asked to carry the burden of significantly increasing projection against the resistance of the skin envelope, it is unlikely to displace and does not need stout suture fixation to underlying alar cartilage. Peck and lobular final contour grafts have been temporarily secured using a percutaneous positioning suture for the Peck graft and three 30 g needles as percutaneous pins, bent at right angles to prevent migration, for the lobular graft (Video 8). The suture and pins are removed at one week followed by strategic taping and “no touch” for 3 months, while we depend on fibrin clot stickiness to help adherence and expect for scar adhesion to eventually hold the tip graft in place (Figs. 2, 3, 4, and 5)

Nonetheless, larger tip grafts can be permanently secured to the domes by placing an interdomal suture as described in this paper and, after tying the knot, leaving the needle and free ends attached and taped out of the way until the end of the procedure at which time the tip graft can be secured as depicted in Video 9.

### Columellar Scar Evaluation

The UC Davis open rhinoplasty scar survey was sent to ASPRS members and 247 responded (Table 1). Despite some expected operator bias, 11% of respondents report that over 25% of their own patient’s scars detracted from the cosmetic result upon intimate observation. In regard to the respondents' revision cases, 32% reported a significant percentage (> 25%), including 9% reporting that the majority (> 50%), of scars done elsewhere were wide, pigmented, notched or otherwise of poor quality. No matter what percentage of columella scars are to any degree a cosmetic negative, there is no chance of an unattractive columella scar if there is no scar.

**Table 1:** SDC supplemental digital content

SDC 1	
A columella scar survey of ASPS membership with questions and response rates. 247 respondents reported: 27/247 (11%) reported that over 25% of their patients were unhappy with columella scar at intimate distance 79/247 (32%) of revision surgeons report a significant incidence (>25%) of the poor-quality columella scars done elsewhere	
1. What % of your patients mention some concern about the final result of the columella scar?	
**9%** of responding plastic surgeons report that the columella scar is a significant patient concern	
91% report <25% of patients mention a concern	
3% report btw 25-50% of patients mention a concern	
6% report >50% of patients mention a concern	
2. What % of your patients do you think the result is diminished to any degree by a columella scar?	
**9%** of responding plastic surgeons report that the cosmetic result is diminished by the scar	
91% report <25% of patients think the result is diminished by scar	
4.5% report btw 25-50% of patients think the result is diminished by scar	
4.5% report >50% of patients think the result is diminished by scar	
3. What % of your patients have on close inspection at their post-op last visit [at least 6 months] a pigmented scar?	
**8%** of responding plastic surgeons report a significant incidence of persistent pigmentation in scar	
92% report <25% of patients have persistent pigmentation	
7.5% report btw 25-50% of patients have persistent pigmentation	
0.5% report >50% of patients have persistent pigmentation	
4. What % of your patients have on close inspection at their post-op last visit [ at least 6 months] a notched scar?	
**8%** of responding plastic surgeons report a significant incidence of scar notching	
92% report <25% of patients have notching	
8% report btw 25-50% of patients have notching	
5. What % of your patients have on close inspection at their post-op last visit [ at least 6 months] a depressed scar?	
**6%** of responding plastic surgeons report a significant incidence of a depressed scar	
94% report <25% of patients have a depressed scar	
6% report btw 25-50% of patients have a depressed scar	
6. What % of your patients have on close inspection at their post-op last visit [ at least 6 months] a wide scar?	
**1%** of responding plastic surgeons report a significant incidence of a wide scar	
99% report <25% have a wide scar	
1% report btw 25-50% have a wide scar	
7. What % of your patients have on close inspection at their post-op last visit [ at least 6 months] a step-off or columella contour flaw?	
**7%** of responding plastic surgeons report a significant incidence of a scar step off or contour flaw	
93% report <25% have a scar step or contour flaw	
7% report btw 25-50% have a scar step or contour flaw	
8. Despite the scar not being noticeable at casual conversational distances, what % of scars would be noticeable at intimate distances?	
**31%** of responding plastic surgeons report a significant incidence of the columella scars being noticeable at intimate distance	
69% report <25% of scars are noticeable at intimate distance	
18% report 25-50% of scars are noticeable at intimate distance	
13% report >50% of scars are noticeable at intimate distance	
9. If you perform secondary rhinoplasty what % of revision patients done elsewhere have scars that have the poor qualities listed above?	
**32%** of responding revising plastic surgeons report a significant incidence of the poor quality columella scars done elsewhere	
68% report < 25% of revision patients present with poor quality columella scars	
23% report 25-50% of revision patients present with poor quality columella scars	
9% report >50% of revision patients present with poor quality columella scars	
10. What % of the time do you feel that patients are unhappy with their open rhinoplasty scar from an intimate distance?	
**11%** of responding plastic surgeons report a significant incidence of patients unhappy with scar when intimate	
89% report <25% of patients unhappy with scar when intimate	
9% report 25-50% of patients unhappy with scar when intimate	
2% report >50% of patients unhappy with scar when intimate	
11. What % of the time do you feel that patients are unhappy with their open rhinoplasty scar from a social distance?	
**5%** of responding plastic surgeons report a significant incidence of patients unhappy with scar at social distance	
95% report <25% of patients unhappy with scar at social distance	
4% report 25-50% of patients unhappy with scar at social distance	
1% report >50% of patients unhappy with scar at social distance	

## Discussion

Regardless of approach, rhinoplasty requires intense perioperative planning to safely achieve the desired esthetic result while protecting or improving the airway and adhering to patient care guidelines [22]. One must always be cognizant of the fact that there are, not only varied global and ethnic proclivities as to what constitutes a desired esthetic result, but also personal preferences within any given culture. These preferences may differ from the surgeon’s sense of the ideal cosmetic result.

Although open rhinoplasty provides the surgeon excellent access and a more comfortable view, the major advantage of open rhinoplasty to the patient is suture restructuring of the nasal tip. To lower and in-fracture the nasal bony dorsum with osteotomes and rasps, the closed exposure is quite adequate. Reduction of the cartilaginous midvault is similar whether accessed by open versus closed rhinoplasty. The same can be said for dorsal augmentation. Closing the midvault, with or without spreader or auto-spreader grafts, can be more challenging, but the exposure is adequate through the endonasal approach. The major long-term advantage of closed structure rhinoplasty is the avoidance of a columella scar. The techniques herein described enable a surgeon to avoid the scar and still provide the restructuring of the nasal tip that has been the primary advantage of open rhinoplasty.

The support of the tip of the nose is naturally the tripod and often this three-pronged support can endure the various manipulations of the lateral and medial crura and domes and still sufficiently maintain tip support [23]. If further tip support is needed, a medial or middle crura strut has been used to strengthen the medial leg of the tripod. Although not done in my practice, the lateral crura component of tip support can be strengthened by placing sutures to repair the disruption of the scroll ligament complex that is caused by the intercartilaginous incision. The ideal angle between the upper lateral cru and the lower lateral cru should be 100 degrees, and this can be approximated with the Cakir resting angle suture [24]. If the tip support from the lateral crura needs further help, lateral crura struts can strengthen or completely restore lateral leg tripod support, as so elegantly shown by Toriumi [25].

The concept of needing a tripod for tip support changes however, if the tip gains sufficient support independent of the strength of the lateral crura. This can most often be achieved by suturing a septal lengthening graft [SEG] not only between the medial crura, but also between the middle crura thus strengthening the domal complex. If the caudal end of the septum is sufficiently long and straight, the sandwich, or tongue-in groove technique, will not only give great support to the medial crura, but will also support the tip if an intercrural suture (plus or minus a middle crura strut) is placed to ensure strength of the domal complex area. The lateral crura can then be weakened, reshaped, interrupted or even dissected off the underlying vestibular skin and rotated into ala pockets as described by Sheen [26]. In each of these scenarios, the lateral crura are no longer paramount for tip support, but they need to be left strong enough to prevent collapse of the tip side walls.

The closed approach and the techniques described in this paper are used with every case regardless of global ethnic variations in both structure and skin type and with consideration for global differences in desired esthetic outcome (Figs. 2, 3, 4, 5, 6, 7, and 8). The closed approach and these techniques are used with every revision rhinoplasty and any prior open rhinoplasty scar is disregarded [unless cosmetically revised] and there have been no incidences of vascular compromise (Figs. 3 and 7).

Although the classic delivery of each alar complex through separate nostrils is our primary approach, Guerrerosantos in 1990 and Holmstrom in 1996 described delivering both domes through one nostril without a columella incision [27, 28]. Bravo furthered this concept of a closed-open approach, and Kamburoglu and Kayikcioglu achieved superior results introducing their variation of closed-open rhinoplasty with extended intranasal incisions [29, 30]. Tezel and Ersoy demonstrated success with their caudal septal graft technique via the closed approach [31]. The closed approach is having a resurgence as innovative surgeons are demonstrating that preservation rhinoplasty, with or without the use of ultrasonic piezotomes, can be done through the closed approach [14, 32–34]. Kosins performs his preservation rhinoplasties closed, if the tip is over projected and not complex, but he uses the open approach, if the tip is complex and needs augmentation [32]. Perhaps the tip techniques herein described could be utilized to allow closed rhinoplasty treatment of some of those complex tips during preservation rhinoplasty.

The endonasal approach to placement of a septal extension type graft was published in by Margulis and Harel in 2007, and the technique was modified and elegantly improved over ensuing years [35, 36]. An ingenious approach to the difficult maneuver of placing an end-to end SEG directly to the caudal end of the septum via the closed approach was described by Scattolin [37]. His transcutaneous sutures could act as a helpful adjunct to the placement of an end-to-end SEG after first securing the SEG to the middle crura, using our technique described above and demonstrated in Videos 2 and 4. Tezel and Ersoy point out that, as opposed to large grafts that lower the entire columella, the batten-to-tip SEGs, similar to the ones portrayed in our videos, could result in retraction of the posterior columella [31]. This is a real concern and, if this posterior retraction is anticipated, it can be prevented by using a larger SEG. If large graft material is not available, one can place smaller pieces of cartilage into the posterior membranous septum to prevent retraction.

Our paper uniquely describes and visually demonstrates an original technique using a caliper to meticulously secure the SEG with intercrural sutures first to the middle crural/domal area and then to the septum. The senior author has found the SEG as described in this paper to be ubiquitous and it is used for cases that need significant increase in tip projection or significant change in tip rotation and, certainly, for all short noses and most Asian and many thick-skinned ethnic noses that need increased tip tension to achieve narrowing. Septal extension grafts in endonasal rhinoplasty are versatile, reversible and reliable [38]. It is remarkable with our technique to be able to confidently hold the tip, which is already secured to the SEG, in the ideal position and “Nail” it in place by suturing the SEG to the septum. Final tip grafts are placed primarily to “fine-tune” the result.

In regard to the columella scar, many patients are not aware there is an alternative to a columella scar, and this may factor into why patients may not complain. Constantian reported that 81% of open rhinoplasty revision complaints were columella related deformities [39]. The senior author’s personal experience and the UC Davis survey indicate that, contrary to the tendency to ignore the issue or minimize its existence, the occurrence of columella scar deformity is real and can be significant. Although columella incisions performed by the many excellent rhinoplasty surgeons can be essentially undetectable, there may be a certain percentage of scars that, even in their best hands, are noticeable and could have been avoided. Scars seen at intimate distances need to be recognized as having some impact. Certainly, the overall gestalt of the nose trumps a scar, but a good result with no scar trumps the same with a scar.

## Summary

Closed rhinoplasty had been relegated to a reductive procedure, but essentially all augmentation and restructuring procedures can be done through the closed approach. This paper demonstrates how to restructure through the standard delivery technique of closed approach. Fixating a septal extension graft initially to the middle crura and domes and secondly to the septum with our closed technique is a very versatile tool to control the tip. The columella scar is often not innocuous, and the columella has a beautiful contour that should be preserved whenever possible.

## Supplementary Information

Below is the link to the electronic supplementary material.

Supplementary file1 (MP4 126748 KB)

Supplementary file1 (MP4 126748 KB)

Supplementary file2 (MP4 154954 KB)

Supplementary file3 (MP4 149217 KB)

Supplementary file4 (MP4 167854 KB)

Supplementary file5 (MP4 177071 KB)

Supplementary file6 (MP4 195529 KB)

Supplementary file7 (MP4 100816 KB)

Supplementary file8 (MP4 148282 KB)

## Declarations

### Human and Animal Participants

This article does not contain any studies with human participants or animals performed by any of the authors
